# Brain metastases from hepatocellular carcinoma: clinical features and prognostic factors

**DOI:** 10.1186/1471-2407-12-49

**Published:** 2012-02-01

**Authors:** Xiao-Bing Jiang, Chao Ke, Guan-Hua Zhang, Xiang-Heng Zhang, Ke Sai, Zhong-Ping Chen, Yong-Gao Mou

**Affiliations:** 1 State Key Laboratory of Oncology in South China and Department of Neurosurgery, Sun Yat-sen University Cancer Center, No. 651, Dong Feng Road East, Guangzhou 510060, People's Republic of China

**Keywords:** Brain metastasis, Hepatocellular carcinoma, Prognosis, Chinese

## Abstract

**Background:**

Brain metastases (BM) from hepatocellular carcinoma (HCC) are extremely rare and are associated with a poor prognosis. The aim of this study was to define clinical outcome and prognostic determinants in patients with BM from HCC.

**Methods:**

Between January 1994 and December 2009, all patients with HCC and BM treated in Sun Yat-sen University Cancer Center were retrospectively reviewed. Univariate and multivariate survival analyses were performed to identify possible prognostic factors.

**Results:**

Forty-one patients were diagnosed with BM from HCC, an incidence of 0.47%. The median age at diagnosis of BM was 48.5 years. Thirty-three patients (80.5%) developed extracranial metastases at diagnosis of BM, and 30 patients (73.2%) had hepatitis B. Intracranial hemorrhage occurred in 19 patients (46.3%). BM were treated primarily either with whole brain radiation therapy (WBRT; 5 patients), stereotactic radiosurgery (SRS; 7 patients), or surgical resection (6 patients). The cause of death was systemic disease in 17 patients and neurological disease in 23. Patients in a high RPA (recursive partitioning analysis) class, treated with conservatively and without lung metastases, tended to die from neurological disease. Median survival after the diagnosis of BM was 3 months (95% confidence interval: 2.2-3.8 months). In multivariate analysis, the presence of extracranial metastases, a low RPA class and aggressive treatment, were positively associated with improved survival.

**Conclusions:**

BM from HCC is rare and associated with an extremely poor prognosis. However, patients with a low RPA class may benefit from aggressive treatment. The clinical implication of extracranial metastases in HCC patients with BM needs further assessment.

## Background

Hepatocellular carcinoma (HCC) represents one of the most common causes of cancer related deaths worldwide [1]. The incidence of HCC demonstrates a striking geographic variability, with the highest rates in East and South-East Asia and Sub-Saharan Africa [1]. It is also one of the top three causes of cancer death in the Asia Pacific region, as a result of the high prevalence of the main etiological agents, hepatitis B virus and C virus infections [2]. In the United States and Europe, where chronic hepatitis C virus infections have been rising, the incidence of HCC is expected to increase further in the next two to three decades [3]. China is an area with epidemic hepatitis B virus, and is estimated to account for half of HCC related deaths worldwide [4].

The presence of brain metastases (BM) is associated with significant morbidity and mortality, and considerable research has focused on improving both survival and quality of life for these patients. BM are most frequently diagnosed in patients with lung, breast and melanoma primaries [5]. However, BM from HCC is extremely rare, with a reported frequency ranging from 0.2% to 2.2% at autopsy [6-9]. Recent therapeutic advances in surgical techniques, including transarterial chemoemobolization (TACE), local ablation, and chemotherapeutic agents, have all contributed to improved survival rates [3]. As a result, the incidence of BM is expected to increase as a result of longer survival for some patients [8]. However, the prognosis for patients with BM is extremely poor, with a median survival of only 1-2 months [7,10,11]. Furthermore, due to its rarity, the identification of prognostic factors and optimal treatment strategies are still being researched. To date, only a few studies from Asia and a small series from America and Europe have been reported [7,8,10-15]. Similar studies from China are lacking. Therefore, in the present study we retrospectively reviewed those patients treated in Sun Yat-sen University Cancer Center (SYSUCC) in China, in an attempt to explore both the clinical characteristics and potential prognostic factors associated with survival in patients with HCC and BM.

## Methods

### Patient population

All patients treated in SYSUCC were prospectively enrolled into a database. Using this database, we identified 10,788 patients diagnosed with HCC between January 1994 and December 2009. Of 8,676 patients with complete follow-up data, 41 developed BM with an incidence of 0.47%, and 7166 patients were detected with hepatitis B virus infection (82.6%), 208 patients with hepatitis C virus infection (2.4%). Diagnosis of HCC was histologically confirmed by surgical resection or by biopsy of the liver mass. The diagnosis in patients without tissue proof was confirmed using the radiological criteria of the presence of a hepatic mass greater than 2 cm identified on one dynamic imaging technique with a typical vascular pattern [3]. The diagnosis of BM from HCC was confirmed by computerized tomography (CT) and/or magnetic resonance imaging (MRI), with or without pathological evidence. Six patients were excluded where the diagnosis was based solely on clinical suspicion, which was not confirmed with imaging evidence. The follow-up period was terminated by death or by the end of the study itself (December 2010). All patients were known to have died at the final follow-up.

### Data collection

All clinical information was retrieved from archived files with the approval of the institutional review board of SYSUCC. Clinical data, including patient demography, clinical presentation, Child-Pugh classification, treatment modality and survival time, was collected. Levels of alpha-fetoprotein (AFP), carcinoembryonic antigen (CEA) and carbohydrate antigen 19-9 (CA19-9) were also reviewed. Patients were assigned to a Radiation Therapy Oncology Group (RTOG) recursive partitioning analysis (RPA) classification following the diagnosis of BM. Four criteria were applied in order to stratify patients: Karnofsky performance status (KPS), primary tumor status, age, and the presence of extracranial metastases. Patients in RPA class I had a KPS ≥ 70, were aged < 65 years, the primary tumor was controlled, and there was no evidence of extracranial metastases. Patients in RPA class III had a KPS < 70. The rest of the patients were in RPA class II [16]. A controlled primary tumor refers to a primary tumor in complete remission after surgical resection or local therapy [7]. The cause of death was determined using the protocol as described by Patchell et al. [17].

### Statistical analysis

Overall survival (OS) was calculated from the radiographic diagnosis of BM until death, or until the date of last follow-up visit for patients who were still alive. Mantel-Cox log-rank test stratified for each factor was applied to compare the Kaplan-Meier curves for survival. Cox proportional hazard model analysis was performed to identify the prognostic factors for OS. Variables with a *P *< 0.05 in univariate analysis were included in the multivariate survival analysis. Analyses were performed using SPSS software 16.0 (SPSS, Chicago, IL, US). A *P*-value less than 0.05 was considered statistically significant.

## Results

### Patient population

A total of 41 patients were diagnosed with BM from HCC, comprising 33 males and 8 females. The median age at diagnosis of HCC was 46.5 years (range, 24.5-81 years). Hepatitis B virus infection was detected in 30 patients (73.2%), and no patient was infected by hepatitis C virus. The majority of patients had undergone surgical resection, laser ablation and/or TACE to treat the primary HCC. Additionally, 2 patients received oral sorafenib for 3 months following a diagnosis of lung metastases. Neither, however, showed a response in terms of tumor control. At the diagnosis of BM, the primary HCC was controlled in 16 patients (39%). Additional demographic data relating to the primary tumor is shown in Table 1.

**Table 1:** Characteristics of hepatocellular carcinoma (n = 41)

Characteristic	No.(%)
Median age at diagnosis of HCC (range), years	46.5(24.5-81)

Etiology	

Hepatitis B	30(73.2%)

Without hepatitis	11(26.8%)

Treatment	

Hepatic resection	22(53.7%)

TACE	27(65.9%)

Chemotherapy	6(14.6%)

Radiotherapy	3(7.3%)

Ablation	3(7.3%)

Hepatic transplantation	2(4.9%)

None	3(7.3%)

Primary tumor	

Controlled	16(39%)

Uncontrolled	25(61%)

Child-Pugh's classification	

A	27(65.9%)

B	8(19.5%)

C	6(14.6%)

The median age at diagnosis of BM was 48.5 years (range, 25-82 years). The median interval between diagnosis of HCC and diagnosis of BM was 15 months (range, 0-120 months). Most patients (80.5%) had already developed extracranial metastases at the diagnosis of BM. Lung metastases were the most frequent (75.6%), followed by bone (22%) and adrenal gland (9.8%). Twenty-four patients (58.5%) presented with a single brain metastasis, and 70.7% of brain lesions were supratentorial. The most frequent presenting symptoms were headache, nausea, and motor weakness. Only one patient was asymptomatic, and the diagnosis was made using whole body positron emission computed tomography (PET-CT). Intracranial hemorrhage occurred in 19 patients (46.3%). Patient characteristics are summarized in Table 2.

**Table 2:** Characteristics of brain metastasis (n = 41)

Characteristic	No.(%)
Median age at diagnosis of HCC (range), years	48.5(25-82)

Median interval between diagnosis of BM from HCC (range), months	15(0-120)

Age at diagnosis of HCC (years)	

< 50	23(56.1%)

≥ 50	18(43.9%)

Gender	

Female	8(19.5%)

Male	33(80.5%)

Site of extracranial metastases	

Lung	31(75.6%)

Bone	9(22%)

Adrenal gland	4(9.8%)

other sites	3(7.3%)

Radiological data	

CT	22(53.7%)

MRI	19(46.3%)

Location of brain lesions	

Supratentorial	29(70.7%)

Infratentorial	7(17.1%)

Combinations	5(12.2%)

APF (ng/ml)	

< 400	15(36.6%)

≥ 400	26(63.4%)

CEA (ng/ml)	

< 5	26(63.4%)

≥ 5	5(12.2%)

Unkown	10(24.4%)

CA199 (u/ml)	

< 35	22(53.7%)

≥ 35	10(24.4%)

Unkown	9(22%)

Symptom	

Headache	17(41.5%)

Motor weakness	14(34.1%)

Nausea	12(29.3%)

Mental change	6(14.6%)

Seizure	4(9.8%)

Visual disturbance	4(9.8%)

Cerebellar dysfunction	3(7.3%)

Aphasia	2(4.9%)

None	1(2.4%)

Interval between diagnosis of HCC and BM (months)	

< 12	25(61%)

≥ 12	16(39%)

Number of brain lesions	

1	24(58.5%)

2	4(9.8%)

≥ 3	13(31.7%)

Brain hemorrhage	

Yes	19(46.3%)

No	22(53.7%)

KPS	

90-100	6(14.6%)

80-89	7(17.1%)

70-79	12(29.3%)

60-69	9(22%)

< 60	7(17.1%)

RPA class	

I	1(2.4%)

II	24(58.5%)

III	16(39.0%)

### Treatment

Symptomatic patients with surrounding brain edema generally received intravenous dexamethasone following the diagnosis of BM. Aggressive treatment included surgical resection, stereotactic radiosurgery (SRS), whole brain radiosurgery (WBRT), and systemic chemotherapy. Treatment modalities were diverse and were based on many factors, including the overall general condition of the patient, the extent of the disease, the number and location of brain lesions, as well as issues relating to the discretion of the individual physician and patients' personal preferences. For those in a relatively poor condition and/or refusing aggressive treatment, supportive treatment was undertaken. Treatment modalities for this group are shown in detail in Table 3.

**Table 3:** Treatment modality for the patients (n = 41)

Treatment	No.(%)
Conservative treatment	23(56.1%)
Aggressive treatment	18(43.9%)
Surgical resection alone	4(9.8%)
Surgery → SRS	2(4.9%)
SRS alone	7(17.1%)
WBRT alone	5(12.2%)

Six patients accepted surgical resection, and complete resection was achieved in four. In two patients in whom the tumor recurred, SRS plus WBRT was performed for one patient, and SRS alone for the other. Two patients received subtotal resection followed by adjuvant SRS. Five patients underwent WBRT alone. The most frequently used fractionation schedule was 30 Gy in 10 fractions (dose range 22.5-30 Gy; mean 28.5 Gy, SD = 3.5 Gy). Seven patients received SRS alone, the median maximal and marginal doses of SRS were 34 Gy (range, 30-38 Gy) and 16 Gy (range, 14-20 Gy)..

### Outcome and prognostic analysis

At the final follow-up, all patients had died. Of the 40 patients with a documented cause of death, 23 patients (57.5%) died from the progression of BMs and 17 patients (42.5%) died from the effects of systemic disease. The impact of patient characteristics and treatment modality on the cause of death was assessed (Table 4). Patients in RPA class III or treated conservatively mainly died as a result of progression of BM. In addition, it was observed that patients with lung metastases were more likely to die from progression of systemic lesions.

**Table 4:** Impact of patient characteristics and treatment modality on the cause of death (n = 40)

	**Cause of death**		
**Variables**	**Neurological (n = 23)**	**Systemic** **(n = 17)**	***P*-value**
Lung metastasis			0.026
Yes	14	16	
No	9	1	
Extracranial metastasis			0.107
Yes	16	16	
No	7	1	
Brain hemorrhage			0.538
Yes	12	7	
No	11	10	
Number of BM			0.337
1	15	8	
≥ 2	8	9	
RPA class			< 0.0001
I or II	8	16	
III	15	1	
Treatment for BM			0.024
Steroid only	17	6	
Surgery or WBRT and/or SRS	6	11	

**Figure 1:**
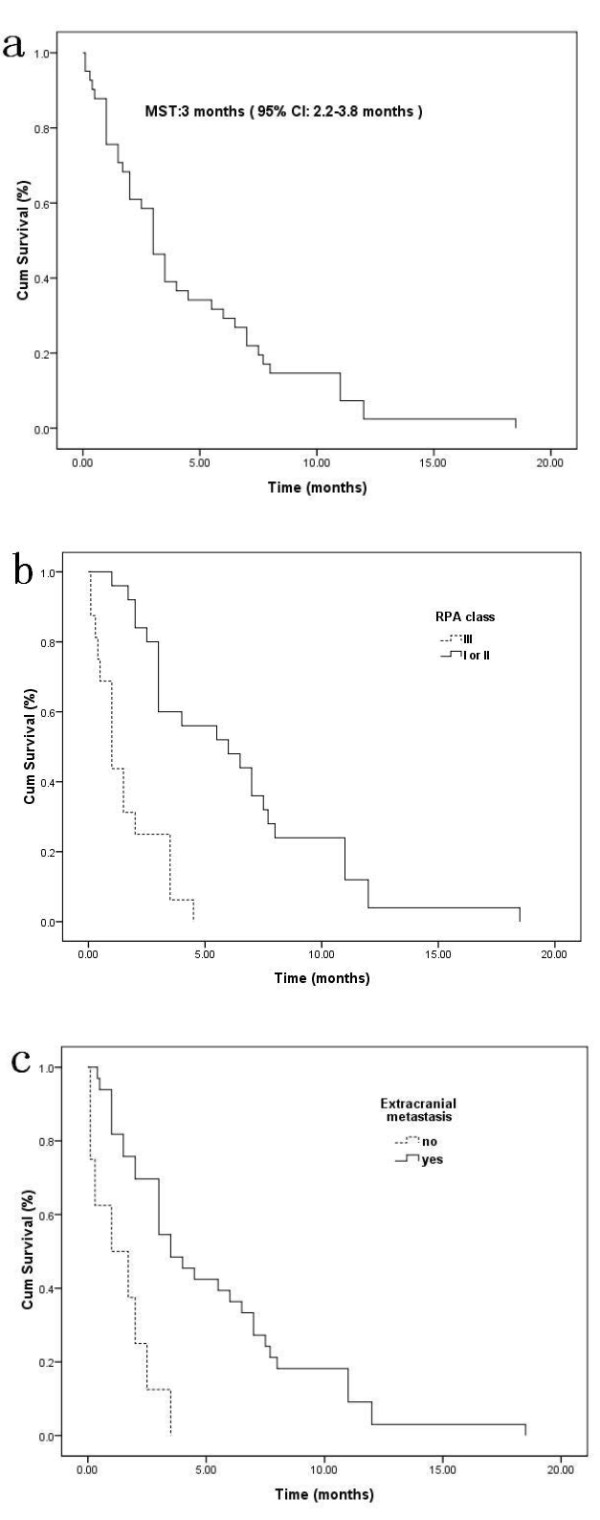
**Kaplan-Meier analyses of overall survival (OS) for 41 patients according to different prognostic factors (overall comparison was performed by log-rank test)**. OS (**a**), OS based on RPA (*p *< 0.0001) (**b**), presence of extracranial metastasis (*p *< 0.0001) (**c**).

The median overall survival time (MST) after diagnosis of BM was 3 months (95% confidence interval: 2.2-3.8 months) (Figure 1a). The 1 month, 6 month and 12 month survival rates were 87.8%, 31.7% and 7.3%, respectively. Tables 5 and 6 demonstrate the results of univariate and multivariate survival analysis, respectively.

**Table 5:** Univariate analysis of survival (n = 41)

	Patients	Mean survival (months)	Median survival (months)	p-Value (log-rank)
Overall survival	41	4.5	3	
Characteristics				
Gender				0.386
Male	33	4.7	3	
Female	8	3.6	2.5	
Age (years)				
< 50	23	4.4	3	0.893
≥ 50	18	4.6	3.5	
Primary tumor				0.156
Controlled	16	5.8	3.5	
Uncontrolled	25	3.7	3	
Etiology				
Hepatitis B	30	4.1	3	0.308
None	11	5.6	3	
Child-Pugh's classification				
A	27	4.9	3.5	0.364
B/C	14	3.8	3	
APF (ng/ml)				
< 400	15	3.4	2	0.218
≥ 400	26	5.1	3.5	
CEA (ng/ml)				
< 5	26	4.8	3	0.725
≥ 5	5	3.9	3	
CA199 (u/ml)				0.455
< 35	22	3.9	3	
≥ 35	10	5.6	3	
Extracranial metastasis				
Yes	33	5.2	3.5	< 0.0001
No	8	1.4	1	
Lung metastasis				
Yes	31	5.4	4	0.001
No	10	1.7	1.7	
Bone metastasis				
Yes	9	5.8	4	0.272
No	32	4.1	2.5	
Brain hemorrhage				
Yes	19	4.1	1.5	0.495
No	22	4.9	3.5	
Interval (months)				
< 12	16	3.5	3	0.272
≥ 12	25	5.1	3.5	
Number of brain lesions				
Single	24	4.2	3	0.572
Multiple	17	4.9	3.5	
Location				
Supratentorial	29	5	3	0.293
Infratentorial or combinations	12	3.4	1.5	
Treatment				
Surgery or WBRT and/or SRS	18	6.8	4.5	0.001
Steroid only	23	2.7	1.5	
RPA class				
I or II	25	6.4	6	< 0.0001
III	16	1.6	1	

**Table 6:** Multivariate analysis of survival (n = 41)

Variable	Hazard ratio	95.0% CI	*P *value
Extracranial metastasis			0.015
Yes	0.337	0.14-0.81	
No	1	----	
RPA class			< 0.0001
I or II	0.169	0.07-0.41	
III	1	----	
Treatment			< 0.0001
Surgery or WBRT and/or SRS	0.28	0.14-0.57	
Steroid only	1	----	

The MST of patients with RPA class I or II was significantly longer than that of RPA class III (6 vs. 1 month, *P *< 0.0001) (Figure 1b). In the multivariate analysis, the RPA class was revealed as the strongest prognostic factor. Patients with extracranial metastasis before or alongside the diagnosis of BM had a longer survival than those without extracranial metastasis (3.5 months vs. 1 month, *P *< 0.0001) (Figure 1c). This relationship remained true in multivariate analysis.

Additionally, patients with lung metastases at the time of diagnosis of BM had a longer survival time compared to those without lung metastasis, although this relationship was no longer significant in the multivariate model. However, there was no survival difference according to gender, age, control of HCC, liver function, levels of AFP, CEA and CA19-9, hepatitis virus B infection, intracranial hemorrhage, location and number of brain lesions, and interval between diagnosis of HCC and BM, in our patient group.

**Figure 2:**
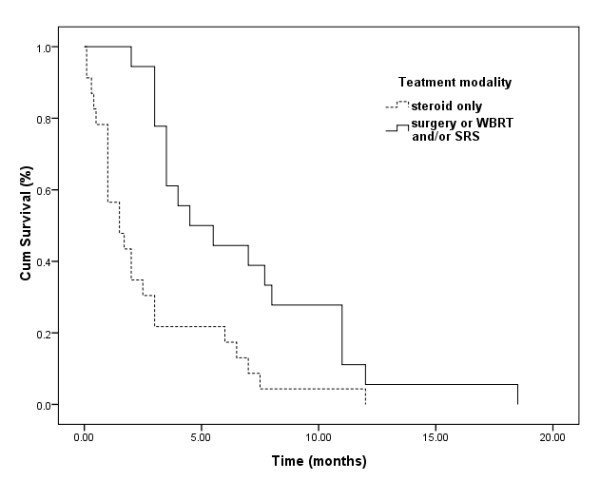
**Kaplan-Meier analyses of overall survival (OS) for 41 patients according to different treatment modalities (overall comparison was performed by log-rank test) (*p *= 0.001)**.

The impact of the treatment modality on overall survival was also evaluated in univariate and multivariate analyses. Aggressive treatment was shown to be associated with a longer survival in univariate analysis (4.5 vs. 1.5 months, *P *= 0.001), which remained an independent prognostic factor in the multivariate model (Figure 2).

## Discussion

With longer survival of patients and enhanced imaging detection techniques, the reported incidence of extra-hepatic metastases in patients with HCC patients is increasing [18]. The most common site for metastases is the lung, followed by regional lymph nodes, bone, the adrenal gland, and occasionally the peritoneum, pancreas, and kidney [18,19]. BM from HCC is a less common occurrence, with various incidence rates reported in the literature. A small series from Korea and Japan reported an incidence of brain metastases between 0.05 and 2.2% [6,8,9,11,20]. The most recent literature from Korea compiled the largest cohort to date, with 62 cases of BM secondary to HCC, an incidence of 0.9% [7]. In our study, the incidence of BM from HCC was 0.47%. However, as has been stated by Choi et al. [7], these figures probably underestimate the true frequency. In our institution, brain scans are not routine for HCC patients. Almost all patients included in this study were diagnosed on the basis of symptoms. As a result, asymptomatic patients with BM may have been missed.

The prognosis in HCC with BM is very poor. As reported in most of the larger studies, overall survival from the time of BM diagnosis is between 1 and 3.7 months [7,10,21]. Our survival result of 3 months concurs with these reports. However, some subsets of patients may benefit from aggressive treatment. In some case reports, survival extended to 12 months after comprehensive treatment [22]. Larger studies have reported a survival time of more than 4 months in patients who received surgery and/or radiotherapy [7,10,11]. The median survival of patients who underwent resection and WBRT can increase to almost 9 months [7]. On the other hand, median survival in patients who received supportive care only was less than 1 month [7,10]. In our study, median survival was 4.5 months for patients who underwent resection and/or radiotherapy, compared to 1.5 months for those treated with supportive care alone. However, not all patients with BM would benefit from aggressive treatment. The identification of prognostic factors would serve to guide the physician into making optimal treatment decisions.

The RPA class has been proposed as an independent prognostic factor for patients with BM from other primary tumors [23,24]. In a study by Choi et al. [7], 62 patients with HCC and BM were included, and patients in RPA classes I and II demonstrated an improved survival when compared to those in RPA class III. However, this relationship was not found to be significant in a further small report [11]. In our study, RPA class was shown to be an independent prognostic factor in multivariate analysis. This result suggests that patients in RPA class I and II may benefit from aggressive treatment.

Patients with extracranial metastases at the time of diagnosis of BM were usually considered to have a high burden of tumor. In patients with BM from other primary tumors, the presence of extracranial metastases has been shown to be associated with a poor prognosis [23,25]. The impact of the presence of BM on survival in patients with HCC remains unclear. In a study by Chung et al. [11], the presence of extracranial metastases was negatively associated with progression free survival. However, this association was not statistically significant. A further study revealed a positive association between median survival and the presence of extracranial metastases [7], although statistical significance was again not achieved. On the other hand, the presence of extracranial metastases prior to a diagnosis of BM was shown to be positively associated with survival in our study. We also found that 87.5% of patients died from progressive BM, whereas only 51.6% of patients with extracranial metastases died from progressive BM. In addition, it was found that patients who did not have lung metastases were more likely to die of progressive brain lesions, which may imply that patients with HCC and BM first, may have tumors that are more poorly differentiated and result in a more aggressive neurovascular invasion. Further studies are needed to elucidate the impact of the presence of extracranial metastases on survival in patients with BM from HCC.

Other important factors may impact on survival of patients with HCC and BM. As the majority of those with HCC have concurrent cirrhosis [3], the activity of the primary tumor and liver function will influence decisions on treatment, and thereby survival. The Child-Pugh classification is used to evaluate the status of liver function, and serum AFP is the most useful tumor marker to reflect tumor burden in HCC [3]. Both parameters have been shown to influence survival in patients with HCC and BM [7]. A recent study demonstrated that a controlled primary tumor was an independent prognostic factor for patients with HCC and extra-hepatic metastases [20]. However, a further retrospective report failed to confirm this association [7]. Additionally, the number of brain lesions is also an important prognostic factor. Many studies have shown that a limited number of brain lesions is associated with a relatively improved survival rate [26], and the median survival of patients with single brain lesions appears longer than that observed in those with multiple lesions [7]. However, we failed to identify any of these parameters as prognostic factors in the present study.

The cause of death for patients with BM from HCC has, to date, been largely unknown. In the present study, the majority of patients were followed up successfully. We explored the relationship between patient characteristics and the cause of death. The results showed that patients in a low RPA class were more likely to die from systemic disease, rather than from progression of BM. Additionally, a properly aggressively treatment had a significant impact on the cause of death, which again confirms its influence on prognosis in patients with BM from HCC. A more rigorous determination of systemic *versus *neurological causes of death could be clarified in a larger study.

Hepatitis B infection is the greatest risk factor for HCC in most geographic areas, with the highest rates in China, Taiwan and Korea [1]. Therefore, similar to previous reports from Korea and Taiwan [7,10,11], the majority of patients in this study had been infected with hepatitis B. The interval between diagnosis of HCC and diagnosis of BM varies in different reports, ranging from 2-54 months [13]. The most recent large series from Korea and Taiwan reported a median interval of 10.5-18.5 months [7,10,11]. Our results were similar, with a median interval between diagnosis of the primary disease and discovery of brain lesions of 15 months. According to previous reviews and reports [7,10,11,13], BM from HCC usually occurs concurrently with a high rate of extracranial metastases. This was confirmed in our study, where 80.5% of patients had concurrent extracranial metastasis. In line with other reports, the lung was the most common site of extracranial metastases (75.6%), followed by bone (22%) [7,10,11,13,18,19]. These results indicate that BM from HCC is indicative of late stage disease, and the presence of systemic metastases should be assessed in HCC patients with BM.

The characteristics of brain lesions described in this study were similar to other reports [7,10,11,13,21]. The distribution of metastasis was supratentorial in 70.7% of our patients compared with 54.8%-83.3% in previous reports [7,10,11,21]. The occurrence of a single metastasis was also similar to earlier studies: 58.5% vs. 50%-66.7%. BM from HCC is frequently associated with hemorrhage as has been reported on many occasions [7,10,11,13,21]. Similarly, 46.3% of patients in our study presented with hemorrhagic brain metastases. Some experts [7] suggest that the hypervascularity of HCC and the underlying coagulopathy due to liver cirrhosis may explain this observation. Hsieh et al. [21] compared the clinical characteristics of patients with and without intracranial hemorrhage, and found that only habitual alcohol consumption was a significant predictor of intracranial hemorrhage in patients with HCC and BM.

## Conclusions

Limited patient samples and its retrospective nature reduce the statistical power of this study. Nonetheless, this first report from a large database in China confirms the clinical characteristics and poor prognosis of patients with HCC and BM. RPA class was identified as an independent prognostic factor, and this association can be applied for the determination of favorable and unfavorable subgroups of patients and optimal treatment strategies. The clinical implications of the presence of extracranial metastases in patients with BM from HCC need further assessment. Our results strongly indicate that sufficiently aggressive treatment may improve prognosis in favorable patient subgroups.

## Competing interests

The authors declare that they have no competing interests.

## Authors' contributions

XJ, CK, and YM participated in the study design. XJ, CK, GZ, XZ, and KS participated in data collection and analysis. XJ, ZC and YM participated in editing and proof reading. All authors read and approved the final manuscript.

## Pre-publication history

The pre-publication history for this paper can be accessed here:

http://www.biomedcentral.com/1471-2407/12/49/prepub
